# Association of Red Blood Cell Distribution Width to Albumin Ratio With the Prevalence of Kidney Stones Among the General Adult Population

**DOI:** 10.1002/iid3.70070

**Published:** 2024-11-21

**Authors:** Linbin Wu, Yuanfeng Zhang, Dake Chen, Wu Chen, Yuanzhao Wu, Bowei Yin, Xianghui Kong, Feilong Miao, Ruxian Ye, Chengpeng Li, Xiaodan Li, Li Chen

**Affiliations:** ^1^ Department of Urology The Wenzhou Third Clinical Institute Affiliated to Wenzhou Medical University, Wenzhou People's Hospital, Wenzhou Maternal and Child Health Care Hospital Wenzhou China; ^2^ Department of Urology Shantou Central Hospital Shantou China; ^3^ Department of Surgical Oncology The Wenzhou Third Clinical Institute Affiliated to Wenzhou Medical University, Wenzhou People's Hospital, Wenzhou Maternal and Child Health Care Hospital Wenzhou China; ^4^ Department of Gynecology and Obstetrics The Wenzhou Third Clinical Institute Affiliated to Wenzhou Medical University, Wenzhou People's Hospital, Wenzhou Maternal and Child Health Care Hospital Wenzhou China

**Keywords:** albumin, kidney stones, NHANES, RAR, RDW

## Abstract

**Background:**

The red blood cell distribution width (RDW) and serum albumin levels are potential indicators of inflammatory conditions. However, the relationship between the RDW to albumin ratio (RAR) and the prevalence of kidney stones in the general adult population is not yet established.

**Methods:**

This study utilized data from the 2007 to 2018 National Health and Nutrition Examination Survey (NHANES) project. RAR levels were calculated by dividing RDW by albumin. Multiple logistic regressions and restricted cubic spline (RCS) regression were applied to examine the associations between RDW, albumin, RAR, and the prevalence of kidney stones.

**Results:**

A total of 31,417 adults (2987 participants with kidney stones) were included for analysis. The mean age of the participants was 47.84 ± 0.23 years, and 48.86% were male. The mean of RDW, albumin, and RAR was 13.25 ± 0.02%, 4.26 ± 0.01 g/dL, and 3.14 ± 0.01, respectively. Compared to the first quartile, the fourth quartile of RDW (OR = 1.44 [1.21–1.72], *P*_trend_ < 0.001) and RAR (OR = 1.62 [1.35–1.95], *P*_trend_ < 0.001) were positively associated with the prevalence of kidney stones, whereas albumin (OR = 0.75 [0.63–0.89], *P*_trend_ < 0.001) was negatively associated with the prevalence of kidney stones after multivariable adjustment. Furthermore, we found that both RDW and RAR levels were positively and non‐linearly related to the prevalence of kidney stones, with inflection points of 13.50% and 3.23, respectively. On the other hand, serum albumin concentrations exhibited a linear association with the prevalence of kidney stones.

**Conclusion:**

Our findings suggest that higher RAR levels are associated with an increased prevalence of kidney stones in the general adult population.

## Graphical abstract

We analyzed the RDW, albumin, and RAR levels obtained from the National Health and Nutrition Examination Survey (NHANES) to investigate the association between RAR levels and the prevalence of kidney stones. We found higher RAR levels are associated with an increased prevalence of kidney stones in the general adult population.

## 1 Introduction

Kidney stones are a prevalent disease of the urinary system, and their incidence and prevalence have been rising alarmingly [1, 2]. This has led to a substantial economic burden worldwide [3, 4]. While kidney stones may range from being asymptomatic to causing significant morbidity and recurrent pain [5, 6], their formation may also trigger the onset of other renal and vascular diseases, including hypertension, chronic kidney disease, and end‐stage renal disease [7, 8]. Several risk factors have been linked to the development of kidney stones, with inflammation recently emerging as a critical factor [9, 10, 11].

Red blood cell distribution width (RDW) is an indicator of the variability of red blood cell volume [12], traditionally used for the differential diagnosis of anemia. However, recent studies have revealed that RDW is a risk factor for coronary heart disease, heart failure, and nephropathy [13, 14, 15], indicating its association with oxidative stress and chronic inflammation [16, 17, 18]. Although the adverse outcomes of RDW have been established in patients with acute kidney injury and chronic kidney failure, its relationship with kidney stones remains unexplored [18, 19]. Serum albumin, a crucial protein in human plasma, helps maintain nutrition and osmotic pressure. Low albumin levels are linked with persistent systemic inflammation [20]. Moreover, evidence suggests that high‐protein diets, while elevating serum albumin, may specifically contribute to the development of kidney stones and other kidney diseases [21].

The RDW to albumin ratio (RAR) is a novel inflammatory biomarker previously used to assess the prognosis of patients with stroke, severe acute pancreatitis, and acute respiratory distress syndrome [22, 23, 24]. Several studies have highlighted the prognostic value of RAR in patients with acute kidney injury [25]. However, the role of RAR in kidney stone patients remains unknown. Therefore, we analyzed the RDW, albumin, and RAR levels obtained from the National Health and Nutrition Examination Survey (NHANES) to investigate the association between RAR levels and the prevalence of kidney stones.

## 2 Materials and Methods

### 2.1 Study Population

The National Health and Nutrition Examination Survey (NHANES) is a program that aims to evaluate the health and nutritional status of adults and children in the United States [26]. The NHANES has been collecting and releasing electronic data from participants on a 2‐year cycle since 1999. All interview and medical examination data can be downloaded from the official website (https://www.cdc.gov/nchs/nhanes). The National Center for Health Statistics (NCHS) Research Ethics Review Board approved the research protocols, and all participants provided written informed consent.

**Figure 1:**
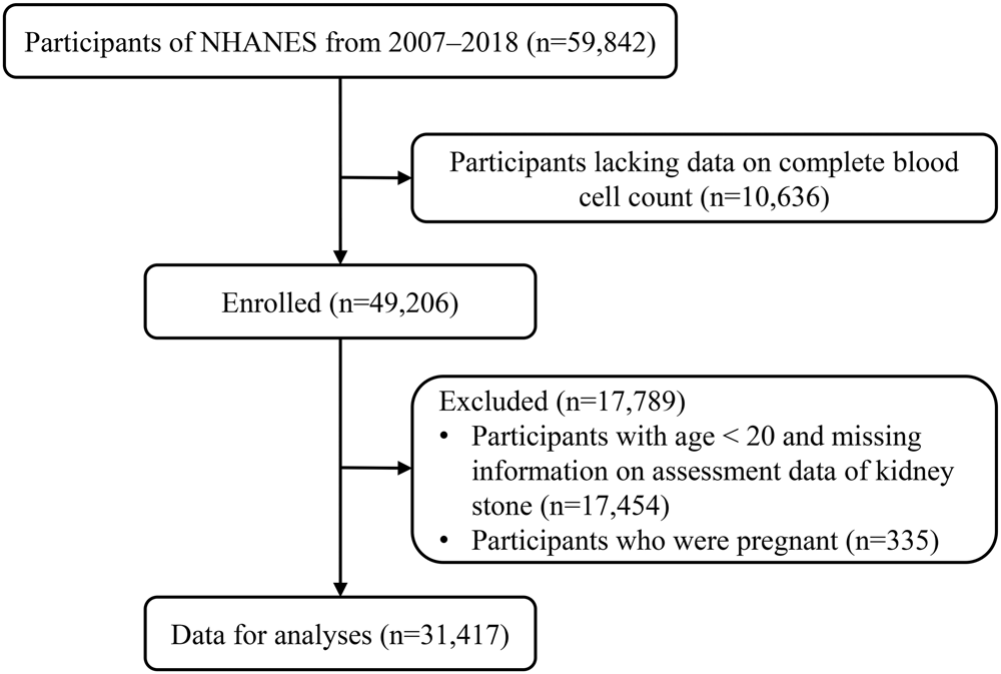
Flowchart of the study participants.

A total of 59,842 individuals participated in the NHANES during 2007–2018. Participants lacking data on complete blood cell count (*n* = 10,636) and participants with age < 20 or missing information on assessment data of kidney stones were excluded (*n* = 17,454). Moreover, pregnant participants were excluded (*n* = 335). Finally, a total of 31,417 participants were included in this study (Figure 1).

### 2.2 Assessment of RAR and Kidney Stones

Blood specimens were collected at the NHANES mobile examination centers (MECs). The RDW was measured as part of the complete blood count (CBC) using the Beckman Coulter MAXM Instrument. This instrument utilizes the Beckman Coulter method for counting, sizing, automatic diluting, and mixing to process the samples [27]. The DcX800 method, employing a bichromatic digital endpoint approach, was employed to determine the serum albumin concentration. This method involves the formation of a complex between serum albumin and the Bromcresol Purple (BCP) reagent [28]. The RAR was calculated as the ratio of RDW to albumin. To explore the exact relationship between these hematology parameters and the prevalence of kidney stones, we treated RAR as a continuous variable and four categorical variables. Kidney stones were defined as a self‐reported doctor's diagnosis obtained by asking the following question: “Has a doctor or other health professional ever told you that you have kidney stones?”

### 2.3 Assessment of Covariates

NHANES provided information about age (years), sex (male or female), race/ethnicity (Mexican American, other Hispanic, non‐Hispanic white, non‐Hispanic black, or other race), education level (below high school, high school, or above high school), body mass index (BMI) categorized as < 25.0, 25.0–29.9, > 29.9 kg/m^2^, serum calcium (mmol/L), serum phosphorus (mmol/L), and estimated glomerular filtration rate (eGFR, ml/min/1.73 m²). Income was measured using the poverty‐income ratio (PIR) and categorized as ≤ 1.0, 1.1–3.0, and > 3.0 [29]. Never smokers were identified as those who reported smoking < 100 cigarettes over the course of their lifetime. Those who smoked > 100 cigarettes in their lifetime were classified as current smokers, and those who smoked > 100 cigarettes and had quit smoking were labeled as former smokers [30]. Drinking status was classified as nondrinker, low‐to‐moderate drinker (< 2 drinks/day in men and < 1 drink/day in women), or heavy drinker (≥ 2 drinks/day in men and ≥ 1 drinks/day in women) [30]. Physical activity was divided into three groups: inactive (no leisure‐time physical activity), insufficiently active (moderate activity 1–5 times per week with metabolic equivalents [MET] 3–6 or vigorous activity 1–3 times per week with MET > 6), and active (those who had more moderate or vigorous activity than the previously mentioned) [31]. We obtained information on the prevalence of hypertension, diabetes, and cancer among participants using self‐reported questionnaires.

### 2.4 Statistical Analysis

The data analysis was performed in compliance with the National Center for Health Statistics recommendations, with consideration of the primary sampling units, sample weights, and strata to obtain reliable national estimates. Weighted analyses were conducted using the “survey” package in R. Normally distributed continuous variables were presented as means with standard errors (SEs), whereas non‐normally distributed continuous variables were presented as medians with interquartile ranges (IQRs). Categorical variables were presented as numbers with percentages. Continuous variables were compared using Student's t‐test (for normal distribution) or Mann–Whitney U test (for non‐normal distribution), while categorical variables were compared using the chi‐square test.

In our study, missing data were minimal for most covariates, with less than 2% missing values. However, drinking status had 8.5% missing, and the poverty income ratio (PIR) had 9.4% missing. To address these missing values, we employed the “mice” package combined with the random forest algorithm for multiple imputations. We incorporated imputed values into our primary models and verified their consistency by comparing them with models that excluded missing data. The estimates from the imputed data aligned with those from the models excluding missing data, confirming the reliability of our imputations.

The RAR was divided into quartiles, with the lowest quartile serving as the reference category. Multiple logistic regression models were employed to determine the adjusted odds ratios (ORs) and 95% confidence intervals (CIs) for the association between the prevalence of kidney stones and continuous RAR or quartiles of RAR. We used the Akaike Information Criterion (AIC) to assess model fit for our regression analyses. Restricted cubic spline regression model analysis with knots at the 10th, 50th, and 90th percentiles of each variable was conducted to investigate the dose–response curves between the RAR and the prevalence of kidney stones. Stratified analyses were also conducted to assess whether subgroup variables (age, sex, race, education level, family PIR, smoking status, drinking status, BMI, self‐reported hypertension, self‐reported diabetes, and self‐reported cancer) had an impact on the association between RAR and the prevalence of kidney stones. All statistical analyses were conducted using R software (version 4.2.0). A two‐sided *p*‐value < 0.05 was considered statistically significant.

## 3 Results

### 3.1 The Characteristics of All Participants at Baseline

In this analysis, a total of 31,417 adults, including 2987 participants with kidney stones, were included (Table 1). The participants had a mean age of 47.84 ± 0.23 years, with 48.86% being male. The mean values for RDW, albumin, and RAR were 13.25 ± 0.02%, 4.26 ± 0.01 g/dL, and 3.14 ± 0.01, respectively. Participants with kidney stones exhibited several distinguishing characteristics compared to those without kidney stones (*p* < 0.001). They were more likely to be older, male, Non‐Hispanic white, nondrinkers, former smokers, obese, and less physically active. Additionally, they had higher RDW, lower albumin, higher RAR, lower serum calcium, lower serum phosphorus, and lower eGFR (*p* < 0.001). Furthermore, participants with kidney stones had a higher prevalence of hypertension, diabetes, and cancer (*p* < 0.001).

**Table 1:** Baseline characteristics of the general adult population in NHANES 2007–2018.

Characteristics	Total	Nonstone formers	Stone formers	*p*‐value
Participants, *N*	31,417	28,430	2987	
Age, years	47.84 (0.23)	47.19 (0.24)	53.66 (0.32)	< 0.001
Male, %	15,351 (48.86)	13,684 (47.87)	1667 (54.67)	< 0.001
Race/ethnicity, %				< 0.001
Mexican American	4752 (15.13)	4363 (8.86)	389 (6.13)	
Other Hispanic	3316 (10.55)	2986 (6.00)	330 (5.10)	
Non‐Hispanic White	12,953 (41.23)	11,335 (65.48)	1618 (76.69)	
Non‐Hispanic Black	6533 (20.79)	6145 (11.37)	388 (5.81)	
Other race	3863 (12.3)	3601 (8.29)	262 (6.27)	
Education level, %				0.937
Below high school	7794 (24.81)	7031 (15.98)	763 (16.05)	
High school	7154 (22.77)	6478 (22.98)	676 (23.26)	
Above high school	16,469 (52.42)	14,921 (61.04)	1548 (60.69)	
Family PIR, %				0.092
≤ 1.0	6984 (22.23)	6351 (15.26)	633 (13.47)	
1.1–3.0	13,297 (42.32)	11,990 (35.98)	1307 (37.39)	
> 3.0	11,136 (35.45)	10,089 (48.76)	1047 (49.15)	
Drinking status, %				< 0.001
Nondrinker	7207 (22.94)	6458 (17.80)	749 (20.69)	
Low‐to‐moderate drinker	21,722 (69.14)	19,656 (72.46)	2066 (72.62)	
Heavy drinker	2488 (7.92)	2316 (9.73)	172 (6.69)	
Smoking status, %				< 0.001
Never	17,483 (55.65)	16,017 (56.25)	1466 (50.25)	
Former	7545 (24.02)	6623 (24.03)	922 30.16)	
Current	6389 (20.34)	5790 (19.72)	599 (19.59)	
BMI status, %				< 0.001
< 25.0	8916 (28.38)	8325 (30.31)	591 (19.56)	
25.0–29.9	10,331 (32.88)	9330 (32.96)	1001 (32.51)	
> 29.9	12,170 (38.74)	10,775 (36.72)	1395 (47.93)	
Physical activity, %				< 0.001
Inactive	8482 (27)	7515 (21.80)	967 (27.62)	
Insufficiently active	9755 (31.05)	8867 (31.97)	888 (30.51)	
Active	13,180 (41.95)	12,048 (46.23)	1132 (41.86)	
Serum calcium, mmol/L	2.35 (0.00)	2.35 (0.00)	2.34 (0.00)	0.030
Serum phosphorus, mmol/L	1.20 (0.00)	1.21 (0.00)	1.18 (0.01)	< 0.001
eGFR, mL/min/1.73 m^2^	94.15 (0.31)	94.87 (0.32)	87.68 (0.47)	< 0.001
RDW, %	13.25 (0.02)	13.23 (0.02)	13.45 (0.04)	< 0.001
Albumin, g/dL	4.26 (0.01)	4.27 (0.01)	4.20 0.01)	< 0.001
RAR, %/g/dL	3.14 (0.01)	3.13 (0.01)	3.23 0.01)	< 0.001
Self‐reported hypertension, %	11,536 (36.72)	10,011 (30.73)	1525 (47.16)	< 0.001
Self‐reported diabetes, %	4256 (13.55)	3572 (9.11)	684 (18.55)	< 0.001
Self‐reported cancer, %	3006 (9.57)	2552 (9.67)	454 (15.92)	< 0.001

*Note:* Normally distributed continuous variables are described as means ± SEs. Categorical variables are presented as numbers (percentages). *N* reflect the study sample while percentages reflect the survey‐weighted.

Abbreviations: BMI, body mass index; eGFR, estimated glomerular filtration rate; RAR, RDW to albumin ratio; RDW, red blood cell distribution width.

### 3.2 Association Between the RAR and the Prevalence of Kidney Stones

Table 2 displays the association between continuous RAR and the prevalence of kidney stones in the general adult population. In the unadjusted model, both continuous RDW and RAR showed positive associations with kidney stone prevalence, while continuous albumin displayed a negative association. These relationships persisted even after adjustment in model 2. In model 3, continuous RDW (OR = 1.09 [1.04–1.13], *p* < 0.001) and RAR (OR = 1.31 [1.16–1.47], *p* < 0.001) remained positively correlated with kidney stone prevalence, whereas continuous albumin (OR = 0.71 [0.59–0.87], *p* < 0.001) exhibited the opposite association.

**Table 2:** Multiple logistic regression associations of continuous red blood cell distribution width to albumin ratio (RAR) with the prevalence of kidney stones among the general adult population in NHANES 2007–2018.

	RDW (%)		Albumin (g/dL)		RAR (%/g/dL)	
	OR (95% CI)	*p*‐value	OR (95% CI)	*p*‐value	OR (95% CI)	*p*‐value
Crude	1.12 (1.09–1.16)	< 0.001	0.55 (0.48–0.64)	< 0.001	1.49 (1.36–1.64)	< 0.001
Model 1	1.13 (1.09–1.17)	< 0.001	0.55 (0.47–0.64)	< 0.001	1.55 (1.39–1.73)	< 0.001
Model 2	1.09 (1.04–1.13)	< 0.001	0.71 (0.59–0.87)	< 0.001	1.31 (1.16–1.47)	< 0.001

*Note:* Model 1 was adjusted for age (continuous), sex (male or female), and race/ethnicity (Mexican American, Other Hispanic, non‐Hispanic White, non‐Hispanic Black or Other); model 2 was adjusted as model 1 plus education level (below high school, high school, or above high school), family poverty income ratio (< 1.0, or ≥ 1.0), smoking status (never smoker, former smoker, or current smoker), drinking status (nondrinker, low‐to‐moderate drinker, or heavy drinker), BMI ( < 25.0, 25.0–29.9, and > 29.9), physical activity (inactive, insufficiently active, or active), serum calcium (continuous), serum phosphorus (continuous), estimated glomerular filtration rate (continuous), self‐reported hypertension, self‐reported diabetes (yes or no), self‐reported cancer (yes or no).

The associations between RAR quartiles and kidney stone prevalence were also analyzed (Table 3). The crude model revealed positive correlations between RDW, RAR, and kidney stone prevalence, as well as a negative correlation between albumin and kidney stone prevalence. After adjusting for age, sex, and race, these relationships remained statistically significant. Compared to the first quartile, the fourth quartile of RDW (OR = 1.44 [1.21–1.72], *P*_trend_ < 0.001) and RAR (OR = 1.62 [1.35–1.95], *P*_trend_ < 0.001) were significantly and positively associated with kidney stone prevalence, after accounting for all confounding factors. Conversely, albumin (OR = 0.75 [0.63–0.89], *P*_trend_ < 0.001) exhibited a significant negative association with kidney stone prevalence.

**Table 3:** Multiple logistic regression associations of quartiles of red blood cell distribution width to albumin ratio (RAR) with the prevalence of kidney stones among the general adult population in NHANES 2007–2018.

	Quartile 1	Quartile 2	Quartile 3	Quartile 4	*P*_trend_
	OR (95%)	OR (95%)	OR (95%)	OR (95%)	
RDW (%)					
Range	< 12.50	12.50–13.10	13.11–13.90	> 13.90	
Crude	1.00 [Reference]	1.41 (1.19–1.67)	1.61 (1.40–1.86)	1.86 (1.58–2.19)	< 0.001
Model 2	1.00 [Reference]	1.33 (1.13–1.57)	1.45 (1.25–1.69)	1.77 (1.49–2.10)	< 0.001
Model 3	1.00 [Reference]	1.29 (1.09–1.52)	1.31 (1.13–1.52)	1.44 (1.21–1.72)	< 0.001
Albumin (g/dL)					
Range	< 4.00	4.00–4.20	4.21–4.50	> 4.50	
Crude	1.00 [Reference]	0.84 (0.72–0.97)	0.71 (0.63–0.80)	0.58 (0.50–0.67)	< 0.001
Model 2	1.00 [Reference]	0.81 (0.69–0.94)	0.68 (0.60–0.77)	0.58 (0.50–0.68)	< 0.001
Model 3	1.00 [Reference]	0.89 (0.76–1.04)	0.80 (0.70–0.93)	0.75 (0.63–0.89)	< 0.001
RAR (%/g/dL)					
Range	< 2.88	2.88–3.10	3.11–3.41	> 3.41	
Crude	1.00 [Reference]	1.42 (1.23–1.63)	1.71 (1.49–1.96)	2.06 (1.79–2.38)	< 0.001
Model 2	1.00 [Reference]	1.36 (1.18–1.56)	1.61 (1.39–1.87)	2.10 (1.79–2.45)	< 0.001
Model 3	1.00 [Reference]	1.26 (1.09–1.47)	1.38 (1.18–1.62)	1.62 (1.35–1.95)	< 0.001

*Note:* Model 1 was adjusted for age (continuous), sex (male or female), and race/ethnicity (Mexican American, Other Hispanic, non‐Hispanic White, non‐Hispanic Black or Other); Model 2 was adjusted as model 1 plus education level (below high school, high school, or above high school), family poverty income ratio (< 1.0, or ≥ 1.0), smoking status (never smoker, former smoker, or current smoker), drinking status (nondrinker, low‐to‐moderate drinker, or heavy drinker), BMI ( < 25.0, 25.0–29.9, and > 29.9), physical activity (inactive, insufficiently active, or active), serum calcium (continuous), serum phosphorus (continuous), estimated glomerular filtration rate (continuous), self‐reported hypertension, self‐reported diabetes (yes or no), self‐reported cancer (yes or no).

### 3.3 Dose–Response Associations of RAR With Kidney Stone Prevalence

**Figure 2:**
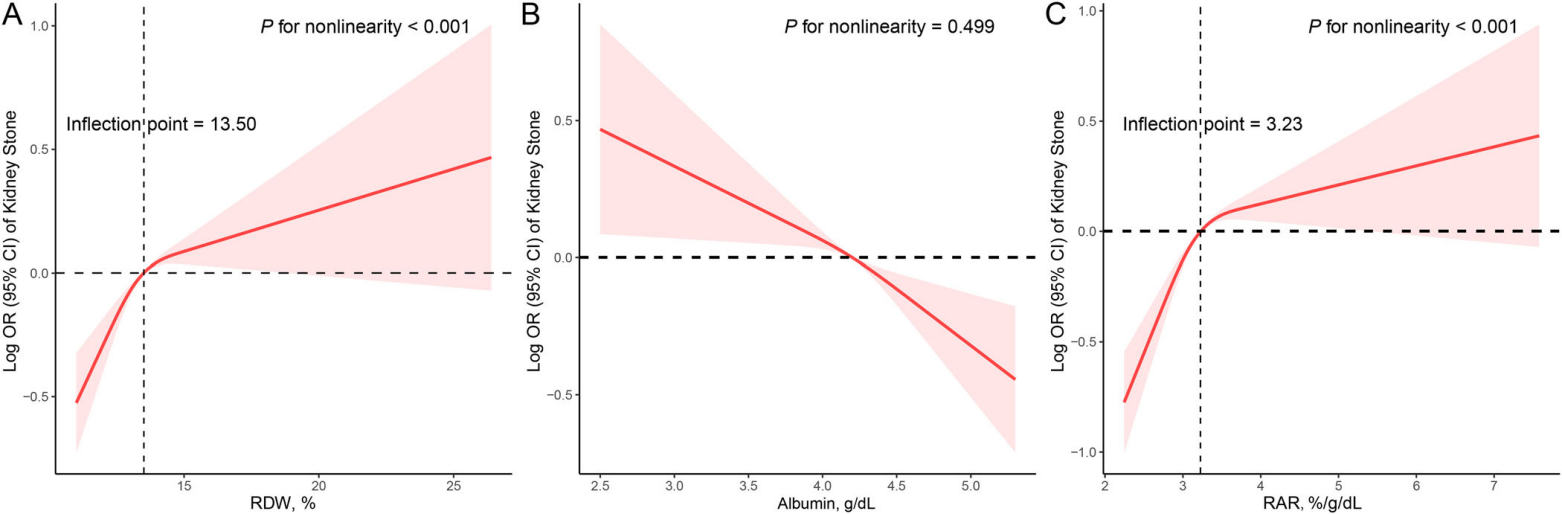
Dose–response associations of the red blood cell distribution width to albumin ratio (RAR) and its components with the prevalence of kidney stones using restricted cubic spline (RCS) analysis (A: RDW and kidney stones; B: Albumin and kidney stones; C: RAR and kidney stones). Analyses were adjusted for covariates age (continuous), sex (male or female), race/ethnicity (Mexican American, Other Hispanic, Non‐Hispanic White, Non‐Hispanic Black or Other), education level (below high school, high school, or above high school), family PIR (< 1.0, or ≥ 1.0), smoking status (never smoker, former smoker, or current smoker), drinking status (nondrinker, low‐to‐moderate drinker, or heavy drinker), BMI (< 25.0, 25.0–29.9, and > 29.9), physical activity (inactive, insufficiently active, or active), serum calcium (continuous), serum phosphorus (continuous), estimated glomerular filtration rate (continuous), self‐reported hypertension, self‐reported diabetes (yes or no), and self‐reported cancer (yes or no).

Figure 2 presents the dose–response associations of RAR with kidney stone prevalence. Both RDW (*P* for nonlinearity < 0.001, Figure 2A) and RAR (*P* for nonlinearity < 0.001, Figure 2C) demonstrated nonlinear and positive associations with kidney stone prevalence. The inflection points were found at 13.50% for RDW and 3.23 for RAR. The OR values for kidney stone prevalence increased rapidly at the beginning of the curves, and after reaching an inflection point, the rate of increase became more gradual, indicating a saturation effect. This suggests that as RDW and RAR values increase beyond a certain threshold, the impact on kidney stone prevalence becomes less pronounced. Conversely, there was a linear and negative association between albumin and kidney stone prevalence (*P* for nonlinearity = 0.499, Figure 2B). As albumin levels increased, the OR values for kidney stone prevalence consistently decreased.

### 3.4 Stratified Analysis

To further investigate the association between RAR and kidney stone prevalence, subgroup analysis was conducted based on age, sex, race, PIR, smoking status, drinking status, BMI, self‐reported hypertension, diabetes, and cancer (Table 4). The results revealed that age played a significant role in influencing this relationship between RAR and kidney stone prevalence (*P* for interaction < 0.001). In the subgroup analysis, individuals younger than 60 years old showed a significant positive relationship between RAR and the prevalence of kidney stones. However, in the group of individuals older than 60 years old, this association was not observed. The other subgroups did not exhibit any significant influence on the association between RAR and kidney stone prevalence.

**Table 4:** Stratified analyses of the associations between quartiles of red blood cell distribution width to albumin ratio (RAR) with the prevalence of kidney stones among the general adult population in NHANES 2007–2018.

Subgroups	*N*	Quartiles of RAR levels, %/g/dL				*P*_trend_	*P*_interaction_
		< 2.88	2.88–3.10	3.11–3.41	> 3.41		
Age, years							< 0.001
< 60	20,616	1.00 (Ref.)	1.49 (1.24–1.79)	1.74 (1.45–2.08)	2.05 (1.68–2.51)	< 0.001	
≥ 60	10,801	1.00 (Ref.)	0.81 (0.61–1.08)	0.83 (0.64–1.08)	1.02 (0.75–1.38)	0.582	
Sex, %							0.160
Male	15,351	1.00 (Ref.)	1.25 (1.03–1.53)	1.37 (1.09–1.73)	1.56 (1.18–2.07)	0.001	
Female	16,066	1.00 (Ref.)	1.16 (0.92–1.46)	1.23 (0.96–1.57)	1.49 (1.18–1.88)	0.002	
Race, %							0.220
Non‐Hispanic White	12,953	1.00 (Ref.)	1.31 (1.08–1.59)	1.48 (1.21–1.80)	1.75 (1.39–1)	< 0.001	
Other	18,464	1.00 (Ref.)	1.08 (0.84–1.37)	1.00 (0.79–1.26)	1.04 (0.77–1.39)	0.980	
Education level, %							0.132
Below high school	7794	1.00 (Ref.)	1.05 (0.75–1.47)	1.28 (0.91–1.78)	1.12 (0.78–1.60)	0.452	
High school	7154	1.00 (Ref.)	1.03 (0.72–1.46)	1.23 (0.83–2)	1.37 (0.87–2.14)	0.132	
Above high school	16,469	1.00 (Ref.)	1.42 (1.18–1.70)	1.45 (1.14–1.84)	1.91 (1.45–2.52)	< 0.001	
Family PIR, %							0.456
≤ 1.0	6984	1.00 (Ref.)	1.25 (0.89–5)	1.63 (1.12–2.38)	1.67 (1.21–2.31)	< 0.001	
1.1–3.0	13,297	1.00 (Ref.)	1.14 (0.88–1.47)	1.18 (0.91–1.54)	1.50 (1.11–2)	0.008	
> 3.0	11,136	1.00 (Ref.)	1.36 (1.11–1.66)	1.46 (1.17–1.84)	1.70 (1.30–2.23)	< 0.001	
Smoking status, %							0.436
Never smoker	17,483	1.00 (Ref.)	1.44 (1.18–1.76)	1.42 (1.10–1.84)	1.65 (1.32–2.06)	< 0.001	
Former smoker	7545	1.00 (Ref.)	1.17 (0.87–1.59)	1.39 (1.01–1.90)	1.75 (1.20–2.55)	0.004	
Current smoker	6389	1.00 (Ref.)	0.99 (0.69–1.42)	1.29 (0.91–1.83)	1.48 (1.02–2.15)	0.024	
Drinking status, %							0.182
Nondrinker	7207	1.00 (Ref.)	1.47 (0.98–2.20)	1.18 (0.82–1.71)	1.51 (1.00–2.27)	0.171	
Low‐to‐moderate drinker	21,722	1.00 (Ref.)	1.27 (1.05–1.54)	1.45 (1.20–1.76)	1.75 (1.41–2.17)	< 0.001	
Heavy drinker	2488	1.00 (Ref.)	0.80 (0.46–1.37)	1.30 (0.76–2.22)	0.88 (0.51–1.49)	0.780	
BMI, kg/m^2^							0.412
< 25.0	8916	1.00 (Ref.)	1.01 (0.72–1.42)	1.13 (0.78–1.63)	1.26 (0.77–2.06)	0.333	
25.0–29.9	10,331	1.00 (Ref.)	1.34 (1.04–1.72)	1.28 (1.00–1.63)	1.47 (1.06–2.05)	0.030	
> 29.9	12,170	1.00 (Ref.)	1.42 (1.05–1.92)	1.71 (1.25–2.33)	1.99 (1.54–2.57)	< 0.001	
Self‐reported hypertension, %							0.523
Yes	11,536	1.00 (Ref.)	1.34 (1.04–1.73)	1.59 (1.19–2.11)	1.69 (1.30–2.21)	< 0.001	
No	19,881	1.00 (Ref.)	1.23 (0.99–1.51)	1.23 (0.99–1.53)	1.61 (1.22–2.11)	0.002	
Self‐reported diabetes, %							0.505
Yes	4256	1.00 (Ref.)	1.29 (1.10–1.51)	1.35 (1.14–1.61)	1.61 (1.31–1.97)	< 0.001	
No	27,161	1.00 (Ref.)	1.11 (0.73–1.69)	1.55 (1.02–2.38)	1.70 (1.09–2.66)	0.005	
Self‐reported cancer, %							0.085
Yes	3006	1.00 (Ref.)	1.23 (1.03–1.46)	1.43 (1.20–1.70)	1.57 (1.30–1.91)	< 0.001	
No	28,411	1.00 (Ref.)	1.55 (0.97–2.47)	1.17 (0.72–1.89)	1.99 (1.22–3.26)	0.026	

*Note:* Data are presented as OR (95% CI) unless indicated otherwise; Analyses were adjusted for covariates age (continuous), sex (male or female), race/ethnicity (Mexican American, Other Hispanic, Non‐Hispanic White, Non‐Hispanic Black or Other), education level (below high school, high school, or above high school), family poverty income ratio (< 1.0, or ≥ 1.0), smoking status (never smoker, former smoker, or current smoker), drinking status (nondrinker, low‐to‐moderate drinker, or heavy drinker), BMI (< 25.0, 25.0–29.9, and > 29.9), physical activity (inactive, insufficiently active, or active), serum calcium (continuous), serum phosphorus (continuous), estimated glomerular filtration rate (continuous), self‐reported hypertension, self‐reported diabetes (yes or no), self‐reported cancer (yes or no) when they were not the strata variables.

## 4 Discussion

In this study, a total of 31,417 adults were included to investigate the potential association between RAR and the prevalence of kidney stones. Our findings indicated that RDW and RAR exhibited a nonlinear and positive association with the prevalence of kidney stones. Conversely, there was a linear negative association between albumin levels and the prevalence of kidney stones. Stratified analysis revealed that age was the only factor that affected these associations. Specifically, RAR levels were not associated with the prevalence of kidney stones in adults older than 60 years. To the best of our knowledge, this is the first study to report an association between RAR and the prevalence of kidney stones among the general adult population.

Recently, RDW has emerged as a novel prognostic factor in various pathophysiological conditions, including renal disease and inflammation [32]. Studies have suggested that high RDW levels may increase the risk of all‐cause mortality in patients with chronic kidney disease (CKD) [19], as well as be independently associated with sepsis‐related acute kidney injury [18]. Therefore, RDW has been considered as a predictive factor for sepsis‐induced acute kidney injury in intensive care unit patients. In our study, we observed a nonlinear and positive association between RDW and the prevalence of kidney stones. Recent recommendations propose the use of RDW in conjunction with other biomarkers, such as serum albumin levels, which are readily available and commonly used. The RAR, calculated as the ratio of RDW to albumin, provides a more comprehensive assessment of the inflammatory process compared to either marker alone [33]. Xu et al. demonstrated that an increase in RAR was associated with an elevated risk of acute kidney injury (AKI) in sepsis patients [34], and it showed promise in predicting all‐cause mortality and the need for renal replacement therapy in critically ill patients with AKI [25]. Although previous studies have explored the potential association between RAR and kidney diseases, none have examined the role of RAR in kidney stone formation. Our study, however, found that higher RAR levels were associated with an increased prevalence of kidney stones, presenting a new diagnostic approach for kidney stone detection.

The association between the red blood cell distribution width to albumin ratio (RAR) and kidney stone prevalence likely reflects the complex interplay of inflammation, oxidative stress, and metabolic disturbances, all of which are involved in kidney stone pathogenesis. RDW is known to increase in response to inflammatory stimulation and has been associated with adverse outcomes in various inflammatory conditions [35, 36]. Inflammatory conditions, common in metabolic disorders, trigger the premature release of larger, immature red blood cells from the bone marrow [37], leading to elevated RDW. In the context of kidney stones, inflammation may play a crucial role in promoting renal epithelial injury, which is a key event in crystal adhesion and stone formation. Oxidative stress, through the generation of reactive oxygen species (ROS) [38], further contributes to this process by damaging renal tubular cells and promoting the crystallization of urinary components such as calcium oxalate. High RDW levels, therefore, may indicate an underlying pro‐inflammatory and pro‐oxidative state that predisposes individuals to kidney stone development.

Serum albumin, on the other hand, is a well‐established negative acute‐phase reactant, with levels decreasing in inflammatory states due to increased microvascular permeability and albumin leakage [39]. Low albumin levels have been associated with poor nutritional status and systemic inflammation, both of which are risk factors for kidney stone formation. Albumin plays a protective role in the kidneys by binding to calcium and preventing the precipitation of calcium salts. Inflammatory conditions that reduce serum albumin may, therefore, enhance the urinary excretion of free calcium and other stone‐promoting substances, increasing the risk of kidney stone formation.

The RAR, as a composite marker of both elevated RDW and decreased serum albumin, likely amplifies these pathophysiological processes. A high RAR reflects a state of heightened systemic inflammation, oxidative stress, and impaired protein metabolism, all of which may contribute to an environment conducive to stone formation. Chronic inflammation not only affects renal epithelial function but may also influence calcium metabolism, leading to increased urinary supersaturation of stone‐forming compounds such as calcium and oxalate. This systemic inflammatory state, reflected in higher RAR levels, could explain the observed association between RAR and kidney stone prevalence.

In addition, we performed a stratified analysis to assess other factors that may influence the outcome. Our results revealed that age played a significant role in influencing this relationship between RAR and kidney stone prevalence. In the subgroup analysis, a notable finding emerged when considering age as a factor. Individuals younger than 60 years old demonstrated a significant positive association between RAR and the prevalence of kidney stones, indicating that higher RAR levels were linked to an increased likelihood of kidney stone occurrence in this age group. This observation suggests that age may be a critical determinant in the manifestation of this association. In contrast, the group of individuals older than 60 years old did not exhibit a significant correlation between RAR and kidney stone prevalence. It is worth noting that the absence of this association in the older age group suggests that factors other than RAR may play a more dominant role in kidney stone formation among individuals in this age bracket. Overall, these findings highlight the importance of considering age as a significant variable when examining the relationship between RAR and kidney stone prevalence, suggesting that the impact of RAR on kidney stone formation may vary across different age groups. Further research is warranted to elucidate the underlying mechanisms and potential age‐specific risk factors contributing to this association.

This study possesses several notable strengths, enhancing the robustness and validity of our findings. Firstly, the utilization of a representative database collected through a standardized protocol ensures that our results are more convincing and reflective of the general population. The inclusion of a large sample size further strengthens the statistical power of our analysis. Secondly, the incorporation of RAR as a composite indicator of inflammation provides a more comprehensive assessment compared to relying on a single biomarker. By considering both RDW and serum albumin levels, we captured the complex interplay between inflammation, erythrocyte dynamics, and kidney stone prevalence. This multifaceted approach adds depth and reliability to our study. Our findings suggest that RDW and RAR, markers typically associated with inflammation and nutritional status, may serve as practical tools for identifying individuals at higher risk of kidney stones. Clinicians could integrate these markers into routine assessments, particularly for at‐risk populations, to inform preventive strategies such as dietary changes and hydration recommendations aimed at reducing stone formation.

Despite these strengths, our study is not without limitations, which should be acknowledged. Firstly, the cross‐sectional design restricts our ability to establish causal relationships between RAR and the prevalence of kidney stones. Future prospective studies are needed to determine the temporal sequence and causality of these associations. Secondly, the generalizability of our findings may be limited to the US population, as our analysis was based on the NHANES database. Caution should be exercised when extrapolating these results to other populations with different demographic, cultural, and environmental characteristics. Efforts to replicate our study in diverse populations would enhance the external validity of our findings. Lastly, although we rigorously controlled for several confounding factors, the possibility of residual confounding due to unmeasured variables cannot be completely ruled out. Further research should aim to explore additional potential confounders and consider their impact on the association between RAR and kidney stone prevalence. Further research is needed to clarify the mechanisms linking RDW and RAR with kidney stone development. Inflammation likely plays a key role, and exploring this connection could provide deeper insights. Longitudinal studies should assess whether these markers predict stone recurrence or progression, while interventional trials could explore whether reducing RDW or RAR through targeted therapies lowers kidney stone risk.

## 5 Conclusion

Our findings suggest a nonlinear and positive association between RAR and the prevalence of kidney stones among the general adult population. The observed association highlights the potential role of RAR as an informative biomarker in understanding kidney stone pathogenesis. Further research is needed to validate these findings and explore the clinical implications of RAR assessment in identifying individuals at risk for kidney stone formation.

## Author Contributions

The authors' responsibilities were as follows—Li Chen: designed the research, and had primary responsibility for the final content; Linbin Wu: conducted analyses and wrote the first draft of the paper; Yuanfeng Zhang, Dake Chen, Wu Chen, Yuanzhao Wu, Bowei Yin, Xianghui Kong, Feilong Miao, Ruxian Ye, Chengpeng Li, and Xiaodan Li: revised the manuscript; and all authors: read and approved the final manuscript and approved the final submitted version.

## Ethics Statement

All participants provided written informed consent and study procedures were approved by the National Center for Health Statistics Research Ethics Review Board.

## Conflicts of Interest

The authors declare no conflicts of interest.

## Data Availability

NHANES data described in this manuscript are available at: https://wwwn.cdc.gov/nchs/nhanes/.
